# Seasonal Variations in Voluntary Intake and ApparentDigestibility of Forages by Goats in the Chinese Altai Mountains

**DOI:** 10.3390/ani12131652

**Published:** 2022-06-27

**Authors:** Alimu Shabier, Greta Jordan, Andreas Buerkert, Ximing Zhang, Eva Schlecht

**Affiliations:** 1 Rangeland Research Institute, Xinjiang Academy of Animal Science, Urumqi 830011, China; alimsabir001@hotmail.com; 2 Organic Plant Production and Agroecosystems Research in the Tropics and Subtropics, University of Kassel, Steinstrasse 19, 37213 Witzenhausen, Germany; gretajordan@gmail.com (G.J.); buerkert@uni-kassel.de (A.B.); 3 Xinjiang Institute of Ecology and Geography, Chinese Academy of Sciences, Urumqi 830011, China; zhxm@ms.xjb.ac.cn; 4 Section Animal Husbandry in the Tropics and Subtropics, University of Kassel and University of Göttingen, Steinstrasse 19, 37213 Witzenhausen, Germany

**Keywords:** alpine pastures, feed intake, forage variability, goats, grazing itineraries

## Abstract

**Simple Summary:**

Grazing animals have a major impact on rangelands through the consumption and trampling of vegetation along with excreta deposition. In turn, forage availability and quality, shaped by environmental and grazing impacts, affect animal performance and health. This study investigated the interaction between amount and nutritional quality of vegetation on offer and animal feed intake on alpine pastures in the Chinese Altai Mountains. To this end, daily grazing routes, vegetation on offer along the pathways, and forage consumption of goats were monitored during spring and the early and late summer season of two consecutive years. Grazing routes were longer in spring than in summer, leading to larger pasture areas utilised in spring. Despite marked differences in vegetation on offer between the two study years, quantitative feed intake did not exhibit seasonal or annual differences, indicating that the goats’ nutrient intake was not restricted on the mountain ranges.

**Abstract:**

Forage availability and quality directly impact animal performance, ultimately affecting productivity and health. This study aimed to understand the interaction between qualitative and quantitative vegetation availability and feed intake of goats on alpine pastures in the Chinese Altai Mountains. The daily grazing routes of three goats from a local herding family were monitored with GPS devices set at a logging rate of 64 s during spring and the early and late summer season in 2013 and 2014. The quantity and quality of vegetation along their grazing routes was determined, and the amount of feces excreted was measured in a total of five goats per season for the indirect determination of the animals’ feed intake. The grazing routes were longer in spring than in summer, leading to larger grazing areas visited in spring. Vegetation on offer ranged from 980 to 2400 kg dry mass per hectare and was similar in the spring and summer seasons but higher in 2013 than in 2014. Feed consumption of forage and nutrients did not significantly differ between seasons and years, respectively, suggesting that the goats’ nutrient intake was not restricted by interannual variability of forage on offer. Regular monitoring of animal numbers and of vegetation quantity and quality on the mountain rangelands can help responsible government agencies to estimate forage offtake of small ruminants in order to timely adjust grazing pressure in the study region.

## 1. Introduction

China’s rangelands cover 400 million hectares of land, which is approximately 40% of its terrestrial area. Thereby, semi-arid areas in the north and west of China account for about 75% of these rangelands [1]. Often, grasslands located in arid, semi-arid, or alpine areas are characterized by low biomass yield and quality—for China, this especially applies to many regions in Xinjiang and Gansu [2]. Across the country, there are over 3.3 million pastoral households and 260 pastoral counties, many of them dominated by mobile groups of nomadic people [1]. Xinjiang Autonomous Region is a major pastoral area in China with 1.16 million pastoralists living in more than 275.000 households [3]. The total grassland area of Xinjiang covers about 32.6 million hectares [4] and accounts for 29% of the national land area [5], whereby not much information is available on biomass yields and grazing impacts for the rangelands of mountainous northwestern China [6].

In the pastoral regions of China, the vegetation of natural rangelands constitutes the main source of feed for sheep and goats [7], but high numbers of animals grazing these pastures threaten their sustainable use [8]. Compared to their respective carrying capacity, there are on average 34% more animals found on China’s rangelands, and in some parts of Xinjiang animal numbers exceed sustainable stocking rates by up to 70% [9]. This leads to the degradation of natural grasslands [10]. Such overgrazing phenomena have been caused by a tripling of livestock numbers in the past 50 years [5]. They are amplified by climate change phenomena, which for the southern Altai Mountains, located in western China, were reported to have led to a decadal increase of 0.54 °C in temperature and 8.9 mm in precipitation during the past 50 years [11], whereas another study [12] reported a 14% increase in the occurrence of warm and dry years since 1875. From both studies, however, it can be concluded that in the future forage biomass yields on Altai rangelands may at best remain at current levels, because raising temperatures, paralleled by increasing precipitation, entail higher evapotranspiration, thereby decreasing the benefits of increasing soil moisture [13].

Independent of its drivers, a declining forage production and inadequate feed supply entail poor livestock productivity [14]. In particular, poor nutrition slows down animals’ growth rates, reduces milk yields, retards the age of reaching sexual maturity, and prolongs the time between subsequent litters [7]. One of the major constraints to balancing the nutrient requirements of small ruminants by actual feed supply is to quantify what and how much the animals ingest on pasture [15]. Since animals freely grazing on natural rangelands select a large variety of plants and plant parts, the digestibility and the nutritional quality of their ingesta is highly variable and difficult to quantify [16]. The composition of the selected diet depends on a number of environmental factors, animal factors, and variables related to the vegetation [17], which all vary over time. To assure adequate nutrition and thus performance of grazing animals, the amount and quality of feed ingested on rangelands must be known. For the context of the Chinese Altai Mountains, only one study [6] has so far adopted the combined livestock–rangeland perspective recommended by von Wehrden et al. [18] and reported detailed data on the forage intake of sheep as affected by biomass offer, biomass quality, and stocking density on alpine summer pastures. The present study aimed at complementing this information by investigating the feed intake of grazing goats in view of forage supply on the mountain pastures, so as to provide a basis for more informed decisions for rangeland utilization.

## 2. Materials and Methods

### 2.1. Study Area

The study was conducted in Qinghe County, Xinjiang Autonomous Region, China during the vegetation periods of 2013 and 2014 (May–September). The long-term (1958 to 2007) precipitation in the town of Qinghe (46°40′28” N, 90°22′59” E, 1253 m a.s.l.) averages 174 mm per year, with an interannual coefficient of variation of 30%. A rain gauge installed at 2400 m a.s.l. measured an annual precipitation (cumulative rain and snowfall) of 188 mm in 2013 and 133 mm in 2014 [6]. Grazing of transhumant herds on Altai spring and summer pastures of Qinghe County is a traditional practice [19]. The day of departure to the pasture areas as well as the date of return of pastoral herds is set by government officials, along with the area to be grazed by each family and the number of animals admitted. The adherence of livestock keepers to these instructions is closely monitored.

The study was carried out on the spring pasture of Qianghan (46°43′42.06″ N, 90°14′19.57″ E, 1500 m above sea level (a.s.l.)) and the summer pasture of Akbulak (47°12′23.62″ N, 090°14′58.20″ E, 2400 m a.s.l.) in the years 2013 and 2014. While the spring pasture is grazed from end of April to early June (about 50 days), the summer pasture is grazed from early July to early September (about 60 days). In consequence, three monitoring seasons were defined per year, namely spring (mid-May to early June), early summer (mid-July to early August), and late summer (late August to early September). Each monitoring time lasted for five days and concentrated on the goats of one local herding family. Prior to the experiment, structured interviews were conducted with 258 herder households in the Altai mountains of Qinghe County [20]. The household whose goats were monitored was chosen because its herd structure, size, and management were very typical for the majority of the interviewed herders. The herd consisted of 358 animals grazed in herd-release mode [21], whereby small ruminants were managed separately from horses and cattle. The herd was only grazing during daytime and always had access to drinking water and salt licks. All animals were local breeds, whereby 85% were sheep, 12% were Cashmere goats, the rest cattle and horses. Since a companion study investigated the feed intake of sheep [6], the present research focused on the goats that were primarily raised for their Cashmere fiber and slaughtered for meat at old age. In each monitoring time, the goats’ grazing itineraries were recorded on days 1 to 3, herbaceous biomass on offer was determined on day 4, and the goats’ fecal excretion was measured on days 1 to 5. On two consecutive days before each data collection period, the studied animals were weighed in the morning (digital electronic hanging scale, range 5–300 kg, accuracy 0.5 kg).

### 2.2. Determination of Grazing Itineraries

To determine their daily grazing routes, from the total of 42 goats in the herd, five growing male animals of middle social rank were chosen, based on the herder’s suggestion. Within a year, the choice of study animals was fixed, whereas it changed from one year to the next. Among the 5 selected goats, 3 received a lightweight GPS collar (GPS PLUS Globalstar, VECTRONIC Aerospace GmbH, Berlin, Germany) which rested around their neck during 5 monitoring days per monitoring time [22]. Every 64 s, the collars recorded the date, time, altitude, latitude, and longitude. At the end of the 5 days, the recorded raw data file was transferred from the collar to a portable computer. Individual GPS positions estimated from only 3 or fewer satellites were removed, after which all data were converted to UTM grid projection (WGS 1984, zone 46N) and processed using the ArcGIS 9.2 software package (ESRI Corp, Redlands, CA, USA); all tracks were merged per season and supposed to represent the movements of the goat herd altogether. A 50 m wide buffer was placed along each side of the merged tracks (100 m buffer in total), from which the daily roamed area was calculated, which was addressed as the daily pasture area used by the whole herd during the 5-day monitoring period. This area was divided by the total time animals spent on pasture on each of the 5 days, to calculate the area of pasture visited in one day.

### 2.3. Determination of Biomass Offer and Quality

The amount of herbaceous biomass encountered along the goats’ daily grazing itinerary was determined on day 4 at each monitoring time. At intervals of 0.5 km along the itinerary, a sampling quadrat sized 0.25 cm^2^ was placed on the ground and its geographical position was taken with a handheld GPS. The area within the frame was first inspected for area of bare soil, percentage of stone cover, and total vegetation cover. Although we did not attempt to identify different plant species, because the study focused on the nutritional value of the overall rangeland vegetation, a label was assigned to the plot as a whole according to the dominant species groups, namely grasses only, dicotyledonous herbaceous plants (herbs) only, grasses and subshrubs, or grasses and herbs. Subsequently, the height of the vegetation within the 0.25 cm^2^ plot was taken with a ruler, thereby measuring the highest and the shortest patch within the sampling frame as well as one patch considered to represent average height, and calculating the mean height per plot. Lastly, all plants growing within the frame were harvested by hand-cutting at 1 cm height above ground. The resulting mixed plant sample was weighed fresh (battery-driven field scale, range 0–3000 g, accuracy 1 g), placed into cotton bags and air-dried in the shade. When completely dry, samples were weighed again and stored at room temperature until analysis.

### 2.4. Determination of Feed Intake and Digestibility

To determine the amount and quality of the diet ingested by the grazing goats, the total fecal mass excreted per day was measured during the 5 days when GPS tracking took place. The 5 male goats (including those animals wearing a GPS collar) were each fitted with a fecal collection bag [23] on day 1 of each monitoring time. The bags were removed on day 5 but were emptied every 8 to 12 h during this period. Each time the bags were emptied, the amount of fresh feces was determined by weighing on a battery-powered portable scale (range 0–3000 g, accuracy 1 g). Then, the fecal material was transferred into a cotton bag and air-dried in the shade. Once completely dry, all samples were weighed again and pooled per animal and monitoring time. They were stored air dry at ambient temperature until laboratory analysis.

### 2.5. Chemical Analysis of Biomass and Feces Samples

In the laboratory, all biomass samples (*n* = 19 in 2013; *n* = 29 in 2014) were oven-dried at 60 °C for 24 h and ground through a 1 mm screen (FOSS sample mill, CyclotecTM 1093, Haan, Germany). Adhering to standard procedures [24], the percentage of dry matter (DM) was determined by drying at 105 °C for 4 h (method MB3-3.1), and the concentration of organic matter (OM) after overnight combustion at 550 °C (method MB3-8.1). Applying the standard method [25] and some modifications [26], the samples’ content of neutral detergent fiber (NDF: hemicellulose, cellulose, lignin, ash) and acid detergent fiber (ADF: cellulose, lignin, ash) were determined with an Ankom 200 Fiber Analyzer (Ankom Technology, Macedon, NY, USA). The nitrogen (N) concentration (MB3-4.1.2) of samples [24] was determined with a C/N-TCD Analyzer (Elementar, Hanau, Germany), and the concentration of crude protein (CP) was calculated by multiplying the N concentration with factor 6.25 [27].

The analysis of the feces samples (*n* = 30) was analogous to that of plant samples in order to determine the concentrations of DM, OM, N, NDF and ADF (see above). From the concentration of CP in feces, the organic matter digestibility (OMD) of the animals’ diet was calculated according to the empirical Equation (1) [28]:

(1) $$y\;=0.899-0.644\times \exp\left(\frac{-0.5774\times \;\mathrm{CPfec}}{100}\right)$$

where y is OMD (%), 0.899, 6.44, and 0.5774 are fixed-effect parameters, and CPfec is the fecal crude protein concentration (g CP kg^−1^ OM).

From OMD (%) and total fecal OM excretion (FOM; g OM d^−1^), the organic matter intake (OMI; g OM d^−1^) was calculated [29] as follows:

(2) y = *100

where y is OMI (g d^−1^).

Based on OMI and the concentration of DM, CP, NDF, and ADF in pasture vegetation, the intake of these constituents was calculated using Equation (3):

(3) y = x*OMI

where y is the intake of a particular component (g d^−1^), x is the forage concentration, in g kg^−1^ OM, of DM, CP, NDF or ADF, and OMI (g d^−1^) is the organic matter intake. Daily intake values of all components were related to the individual animal’s live weight (LW) and expressed per kilogram of metabolic weight (g kg^−0.75^ LW).

### 2.6. Statistical Analysis

Statistical data analyses were performed in SPSS Statistics 22 [30] to determine differences between the three seasons of each year, and between the two years for each season separately. Thereby, dependent variables were itinerary characteristics, biomass yield and quality, feed intake, and fecal excretion. The Shapiro–Wilk test was used to examine residuals which were checked for normal distribution, and homogeneity of variance was assessed by using Levene’s test. Even though not all variables showed normal distribution, independent t-test (comparison of years) and one-way ANOVA (comparison of seasons) were used to determine statistical differences. In the latter case, Tukey’s post hoc test was used to identify the differing means. Recognizing that probabilities obtained by a parametric test on non-normally distributed data are indicative only, significance was declared at *p* ≤ 0.05. If not specified otherwise, results are presented as means followed by their standard deviation (±) or by the standard error of the mean (SEM), respectively.

## 3. Results

### 3.1. Length of Grazing Itineraries and Size of Pasture Areas

**Figure 1:**
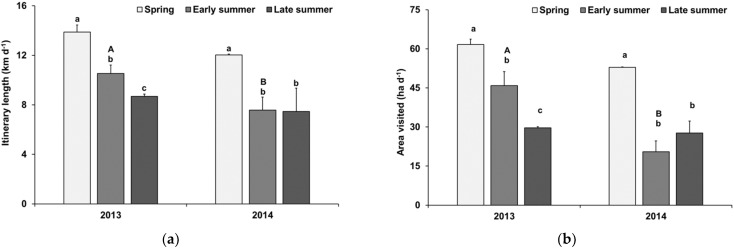
Distance covered by the daily grazing itinerary (**a**) and area of pasture visited (**b**) by the studied goat herd on Chinese Altai Mountain pastures during 2013 and 2014. Small letters (a,b) indicate differences between the seasons of one year; capital letters (A,B) indicate differences between the two years for a given season; *p* ≤ 0.05.

The daily distances covered during grazing varied between seasons and years: 13.9 km d^−1^ (±0.58) was the longest average distance, recorded in spring 2013, whereas the shortest average distance was 7.5 km d^−1^ (±1.88) in late summer 2014. Overall, daily itineraries were longer in 2013 than in 2014 (Figure 1a). However, while in 2013 the length of itineraries differed between all seasons (*p* ≤ 0.05), itinerary length varied closely around 7.5 km (±1.20) in early and late summer 2014. From the buffer zone plotted along the itineraries, a daily grazing area (Figure 1b) of the goat herd of 62 ha d^−1^ (±2.2) and 46 ha d^−1^ (±5.4) was calculated for spring and early summer 2013, respectively, which shrunk to 30 ha d^−1^ (±0.5) in late summer 2013 (*p* ≤ 0.05). In contrast, the area of 28 ha d^−1^ (±4.6) grazed in late summer 2014 was only numerically different (*p* > 0.05) from the area of 21 ha d^−1^ (±4.1) grazed in the early summer of that year.

### 3.2. Quantity and Quality of Pasture Vegetation

**Figure 2:**
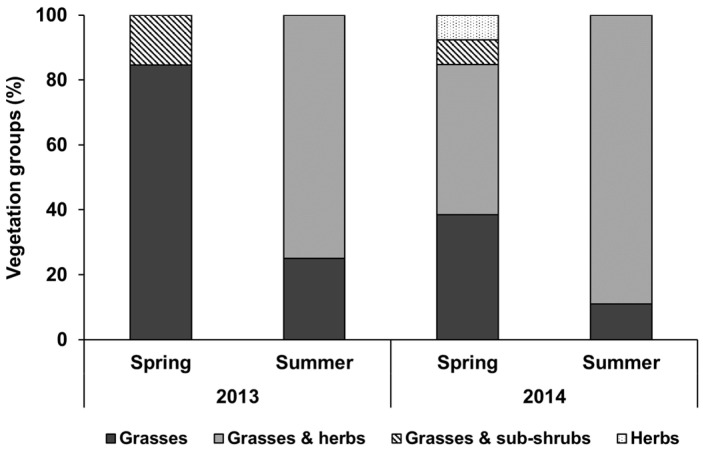
Frequency distribution of plots with different predominant vegetation groups along the itinerary of goats on spring and summer pastures in the Chinese Altai Mountains in 2013 and 2014. Dicotyledonous plants are grouped into herbaceous species (termed herbs) and subshrubs, respectively.

Across the spring and summer season, the amount of pasture biomass along the goats’ grazing itineraries (Table 1) averaged 2276 kg DM ha^−1^ in 2013 and 1122 kg DM ha^−1^ in 2014 (*p* ≤ 0.05). Thereby, biomass DM offer did not differ between the three grazing seasons of each year (*p* > 0.05). The vegetation height in individual plots ranged from 2 cm to 55 cm and was similar across seasons in 2013. With an average height of 20 cm, vegetation was higher (*p* ≤ 0.05) in early summer 2014 compared to the spring (10 cm) and late summer (13 cm) of 2014 (Figure 2). Likewise, with 44% and 32% of the sampled plots exhibiting a vegetation cover of >50% and >75%, respectively, vegetation cover varied little (*p* > 0.05) between seasons in 2013. However, in 2014, the vegetation cover was at 25% on the spring pasture and lower (*p* ≤ 0.05) than on the early- and late-summer pastures, where it averaged 71% (±21.9) across the seasons. Inversely, stone cover was higher (*p* ≤ 0.05) on the spring than on the early- and late-summer pastures of 2014. With 17%, stone cover on the 2014 spring pasture (17%) was about three times higher (*p* ≤ 0.05) than on the 2013 spring pasture, where it was 6%.

The cover contribution of grasses, herbs, and subshrub groups was different across seasons and years (Figure 2): with 85% (2013) and 39% (2014), respectively, the proportion of grasses on spring pastures was higher than on the summer pastures (average 2013: 25%; 2014: 11%). Subshrubs were only found on spring pastures, the summer pastures were dominated by herbs (average 2013: 75%; 2014: 89%).

The nutritional quality of the vegetation on offer along the animals’ grazing itineraries (Table 2) varied between seasons and years. At an average of 380 g DM per kg fresh matter, the DM concentration was lowest in early summer 2013 (*p* ≤ 0.05). The DM concentration of OM varied little and on average ranged from 85% to 90% across years. With 136 g kg^-1^ DM, the CP concentration of the vegetation was highest in spring 2013 but declined to 117 g kg^-1^ DM in early summer 2013 (*p* ≤ 0.05). Average CP concentrations of less than 115 g kg^-1^ DM were determined for early and late summer 2014. Averaging 570 g NDF kg^−1^ DM, the concentration of cell wall constituents in pasture plants was higher (*p* ≤ 0.01) in spring 2014 than in spring 2013 and in summer 2014, when values were below 500 g NDF kg^−1^ DM. With >370 g ADF kg^−1^ DM, the highest average dry-matter concentrations of cellulose and lignin (including ash) were determined in spring of 2013 and 2014, decreasing to approximately 300 g ADF kg^−1^ DM and <290 g ADF kg^−1^ DM in summer 2013 and 2014, respectively.

### 3.3. Feed Intake and Fecal Excretion

The live weight of the five goats used to study feed intake varied closely around 60 kg (±11.8) and was similar across seasons and years (*p* > 0.05). OMD of the selected diet as calculated from the fecal crude protein concentration averaged 70% in spring 2013 and increased to 72% (*p* ≤ 0.05) in the late summer of 2013 (Table 3). In 2014, OMD averaged 76% in the early summer as compared to 70% in spring and 72% in late summer (*p* ≤ 0.001).

The intake of DM and OM (DMI, OMI), as calculated from the excreted amount of feces (Table 4) and OMD, was similar across seasons and years (*p* > 0.05). The average maximum intake of dry matter (DMI: 80 g kg^−0.75^ LW) and organic matter (OMI: 75 g kg^−0.75^ LW) occurred in the early summer season of 2014, and average minimum values (DMI: 63 g kg^−0.75^ LW, OMI: 60 g kg^−0.75^ LW) were observed in the late summer season of 2013. The intake of CP (CPI) was also relatively constant across seasons and years (*p* > 0.05): with 9.5 g kg^−0.75^ LW, the highest average CPI was determined in spring 2014 and the lowest average value of 7.9 g kg^−0.75^ LW was recorded in late summer 2013. With an average value of 43 g kg^−0.75^ LW, the intake of NDF was higher (*p* ≤ 0.05) in spring 2014 than in spring 2013 (31 g kg^−0.75^ LW), but during the summer, no differences in NDF intake were observed throughout the study. Seasonal intake values of ADF varied little in 2013, but in 2014 ADF intake in spring (29 g kg^−0.75^ LW) was higher (*p* ≤ 0.05) than in summer, when intake was below 23 g ADF kg^−0.75^ LW.

The quantitative excretion of feces per goat ranged from 300 to 594 g DM d^−1^ and was not affected (*p* > 0.05) by season and year (Table 4). Similarly, the fecal OM concentration was relatively stable at 78% (±6.9) across seasons and years (*p* > 0.05). With an average value of 3%, the fecal N concentration was higher (*p* ≤ 0.05) in summer 2013 as compared to spring 2013, when a value of 2.7% was determined. The NDF content of feces decreased over time in both years, with the highest values in spring and lowest concentrations in late summer.

## 4. Discussion

The current study was conducted in parallel to three other studies that also dealt with questions of a sustainable management of alpine meadows in the Chinese and Mongolian parts of the Altai Mountains [6,19,31]. In analogy to the approaches used in these studies, we determined daily grazing itineraries of three animals (freely grazed) from position recordings at 64 s intervals with lightweight GPS collars and assessed the amount of herbaceous biomass along the daily tracks. A similar approach, yet for a longer period and with a higher spatiotemporal resolution, was used to understand habitat and forage selection of bison in Kansas, USA [32]. In addition, we used fecal collection bags to measure the amount of fecal mass excreted by five goats during five consecutive days per season. With this information and the fecal N concentration, the animals’ daily feed intake was calculated and related to the amount of forage offered within the area visited during a day on the pasture.

### 4.1. Characteristics of Animals’ Grazing Itineraries

Especially in view of ongoing climate change, mobile livestock husbandry is well-adapted to dryland conditions and has been shown to sustainably support local livelihoods [33]; by enabling pastoralists to flexibly adjust the herds’ grazing location and grazing times to highly variable vegetation [6,34], mobile forms of livestock management allow for an optimal utilization of scare resources. Thereby, the spatiotemporal aspects of livestock movements are to a large extent governed by the availability, quality, and spatial distribution of forage resources [35]. In contrast to goats in Odisha, India, whose daily grazing itineraries closely varied around 6.3 km across seasons [36], the length of daily grazing routes in the present study varied between the seasons and the years. In the spring season of both 2013 and 2014, the daily routes were longer than in the early and late summer seasons, whereby forage offer was either higher or comparable between spring and the (early) summer season. This is in accordance with reports from the Hajar Mountains in Oman where goats walked further distances when forage availability was higher as compared to seasons with lower forage offer [22]. Although seemingly contradictory, such an observation may be explained by the fact that with decreasing forage availability, energy expenditure for walking in search of forage increases with uncertain chances for counterbalancing this loss by adequate feed intake [31], inciting the animals to increase resting and reduce walking activities [21]. In addition, more pleasant weather conditions in spring than in summer may also encourage walking activities of grazing animals [37]. Except for the early summer season of 2013, the length of grazing itineraries in a given season was not different between the two years; this may have been due to the fact that the goats grazed within the same larger area in both years. The mean walking distances of 8 to 13 km d^−1^ were only slightly shorter than the distances of 8 to 14 km d^−1^ reported for Arbas Cashmere goats in Inner Mongolia, China [38], and for goats in the southern Altai Mountains of Mongolia [31], but longer than the 4.7 to 10.2 km d^−1^ walking distances reported for goats from India [36] and for small ruminants grazing harvested fields in the cotton-growing zone of Xinjian [10], as well as of sheep grazing natural grasslands in Inner Mongolia, China [39]. Since the daily grazed area was derived from the itinerary length, interseasonal and interannual differences in the itineraries translated to differences in areas grazed, ranging from 27 to 60 ha per day on average. The latter values were substantially lower than the daily average area of 71 ha grazed by goats on spring and summer pastures in the southern Altai Mountains of Mongolia [31], but were within the range of 18 to 68 ha reported for small ruminant herds in our study region [19].

### 4.2. Quantity and Quality of Pasture Vegetation on Offer

The seasonal changes in the amount and quality of forage encountered on pasture are a major limitation to grazing-based livestock production [6]. They are primarily related to climatic variation that, in interplay with grazing management, lead to a variation in the species composition and biomass production on the rangelands [40]. In contrast to observations made in the Tianshan Mountain region of Xinjiang, China [41], the differences in altitude between the spring and summer pastures did not influence the aboveground biomass production in the present study. Biomass production shows a strong positive correlation with precipitation [13], which can explain the nearly twofold biomass offer in 2013 as compared to 2014. According to local weather station records (https://www.uni-kassel.de/forschung/watercope/home accessed on 27 June 2022), the annual precipitation across the three altitudes averaged 188 mm and 133 mm in 2013 and 2014. Especially for the year 2013, these data corresponded very well to the average annual precipitation of 180 mm recorded at Qinghe station during the period 1966–2015 [11]. This indicates that the environmental conditions encountered during the two study years were typical for this region. In both years, the biomass yield on the studied spring and summer pastures was substantially higher than the 600 to 980 kg DM ha^−1^ reported for pastures of the neighboring Bulgan region in the southern Mongolian Altai during the same years [31]. Furthermore, the biomass yield compared well to the 890 to 3800 kg DM ha^−1^ reported from a nearby controlled-grazing experiment with sheep [6] in July 2013 and July 2014.

From the daily area grazed by the goats and the amount of biomass they encountered, a daily forage amount of >100 kg DM per sheep unit [42] in 2013 and of 40 to 50 kg DM per sheep unit (SU) in 2014 can be calculated from the present data. These values are above the average daily herbage allowance of 26 kg DM per SU reported by Jordan et al. [19] but agree with the 33 to 40 kg DM per SU reported for early summer pastures in the southern Mongolian Altai [31]. While the high values in 2013 correspond to the study region’s herbage allowance at low stocking densities of 6 SU per hectare, the much lower values in 2014 were similar to those determined by Lv et al. [6] for stocking densities of 14 to 20 SU per hectare in the same region and year. While the companion study of Jordan et al. [19] suggests that the higher herbage allowance for livestock on spring and summer pastures in the Chinese as compared to the Mongolian part of the Altai is a result of the stringent grazing rules enforced by the local government, the high interannual variability in vegetation on offer support the notion that stocking densities of pastoral herds should be flexibly adjusted to the yearly biomass development on the seasonal mountain pastures [6,19]. A point of uncertainty of the present approach lies in the fact that the herd-released goats and sheep of the study herder did sometimes, but not always, graze together, so it was difficult to determine the exact stocking densities of the grazed areas.

A high nutritional value of the forage consumed on the pasture is crucial for livestock productivity [43]; it is determined by soil and climatic conditions, the individual plant species and even plant fractions ingested, as well as the phenological stage of the plants at the moment of grazing [44]. The nutritional value of the ingested diet is judged by indicators such as its concentrations of CP, NDF, and ADF, and its digestibility [43]. In our study region, the quality of the grazed vegetation showed intra- as well as interannual variation, which was also reported from pastures in Inner Mongolia, China [45]. The interannual variations in vegetation quality, especially, may in part have been due to the above-mentioned differences in rainfall between 2013 and 2014. In each of these years, the CP concentration was higher in vegetation on spring than on summer pastures; this can be explained by a high proportion of mature plant material in vegetation encountered in summer [45]. As plants mature and ambient temperatures increase during the summer season, their concentrations of cell wall constituents (NDF, ADF) and lignin increase, while their CP concentration and digestibility decrease [46]. This phenomenon is also reflected in the present vegetation samples, where the concentrations of CP on one hand and of NDF and ADF on the other hand show opposing trends of decline and increase, respectively, as the year progresses. This leads to a widening carbon (i.e., cell wall) to nitrogen (i.e., crude protein) ratio, which commonly reduces plant digestibility and feed intake [47], whereas a high CP concentration enhances diet digestibility [48]. Overall, the quality of forage on offer along the animals’ itineraries was similar or even slightly superior to the values reported, for the same years, from spring and summer pastures in the southern Mongolian Altai [31].

### 4.3. Feed Intake

In addition to environmental factors and forage quality aspects that influence the feed intake of grazing ruminants, also aspects such as the size, shape, and texture of plants’ leaves and stems have an influence on their foraging behavior [49,50,51,52]. According to the degree of selectivity allowed, the animals will ingest a diet of higher or lower quality, which will influence their productive performance [53]. Surprisingly, the organic matter digestibility of the ingested diet was always lower in spring than in the (early) summer season, a phenomenon also observed in the Mongolian part of the Altai Mountains. This can be explained by the fact that vegetation development at the higher altitudes of the summer pastures is retarded as compared to that on the lower-lying spring pastures, which results in a similar or even better quality of summer pasture vegetation [31]. The daily OM intake of goats of 59 to 74 g kg^−0.75^ LW determined in the present study was lower than the values obtained for goats in Oman [54] as well as for small ruminants grazing harvested fields in Xingjian’s cotton zone [10], but closely matched with intake values of 44 to 86 g OM kg^−0.75^ LW of goats in the Mongolian Altai [31]. A daily DM intake of 63 g kg^−0.75^ LW as determined in spring and late summer 2013 is very close to the average DM intake requirement (62 g kg^−0.75^ LW) recommended for goats [55], and the values obtained for the early summer of 2013, and spring, early and late summer 2014 were even 16 to 28% higher. This underlines that even in the year 2014 with its lower precipitation and biomass yield, the amount of forage available to the goats enabled unrestricted voluntary feed intake [56]. The intake of CP also closely matched goats’ average requirements of 4.2 g CP kg^−0.75^ LW [55]. The ingestion of ADF and NDF intake did not show high intra- and interannual variation, with the exception of differences between spring 2013 and 2014 (NDF intake), and between spring and summer 2014 (ADF intake). Permitting that a biomass yield above 1000 kg DM ha^−1^ does not limit diet selection and feed intake of grazing small ruminants [56], the low variation in the quantitative intake of cell wall constituents may have been due to above-mentioned high (2013) or at least satisfactory (2014) forage offer on the pastures. This enabled the goats to ingest a diet of higher nutritional quality than the average forage on offer, which is in agreement with observations of Zemmelink and t’Mannetje [57] that high feed allowance increases the animals’ options for diet selection and promotes a higher nutritional quality as well as amount of ingested feed.

### 4.4. Excretion of Organic Matter and Nutrients

Quantifying the amount of feces is the most reliable method to calculate the forage intake of ruminants on pasture [58]. Since fecal matter is collected, the straightforward determination of its N concentration and the calculation of the diet digestibility from established empirical equations that can be applied across ruminant species and environments requires only little additional effort [58,59,60]. The correlation between the fecal N concentration and feed organic matter intake is strong and significant for all types of diets [59]; it relies on the principle of proliferation of rumen bacteria with increasing feed digestibility that entails an enhanced fecal N concentration, while at the same time the total amount of fecal matter decreases. Although the DM concentration in forage on spring and summer pastures differed, the DM concentration of the goats’ feces showed little intra- and interannual variation, which agrees with findings of Wang and Li [61]. In contrast, the significant seasonal differences in the fecal NDF concentration point to a variation in the degree of cell wall digestibility [62]. Across the whole study period, the N concentration in feces was always higher than that of forage; this might either be explained by the above-mentioned relationship between increasing concentrations of undigested bacterial protein in feces at increasing diet digestibility or, alternatively, a certain share of undigested forage nitrogen in the feces. Whereas undigested bacterial protein will be decomposed rather quickly after feces have been excreted, undigested plant nitrogen is only slowly released [63]. Such information, however, is only relevant when addressing organic matter and nutrient recycling within pastures and the emission of greenhouse gases from animal excreta [64].

## 5. Conclusions

The present results on the length of goats’ daily grazing itineraries and grazing areas, quantity and quality of vegetation offered on the grazed areas, and feed and nutrient intake on alpine pastures of the Chinese Altai Mountains indicate that goats are amply supplied with forage, crude protein, and digestible organic matter during the spring and summer transhumance period, even in years of below-average rainfall. Companion studies suggest that this positive situation seems to be largely due to stringent grazing rules enforced by the local government. However, a more flexible adjustment of the length of grazing periods and stocking densities in response to the actual vegetation development in a given year is highly recommended, because in years with above-average forage availability, a higher yield of animal products could be realized without jeopardizing the long-term rangeland productivity. Regular well-timed monitoring of vegetation quantity and quality along with mobile herds’ patterns and intensities of grazing on the mountain ranges can therefore support ecologically and economically sustainable pasture utilization in the study region.

## Figures and Tables

**Table 1:** Seasonal pastures in the Chinese Altai Mountains: altitude, dry matter (DM) biomass yield along goats’ grazing itinerary, vegetation height and cover, as well as stone cover. Values depict arithmetic means ± standard deviation.

Year	Season	n	Altitude	DM	Vegetation	Vegetation	Stone Cover *
			(m a.s.l.)	(kg ha^−1^)	Height (cm)	Cover (%)	(%)
2013	Spring	10	1506 ^b^ ± 1.4	2420 ^A^ ± 429.9	15 ± 10.5	56 ^A^ ± 14.8	6 ^B^ ± 15.6
	Early summer	6	2408 ^a^ ± 103.4	2219 ^A^ ± 358.0	14 ± 6.2	67 ± 20.4	0
	Late summer	3	2275 ^B,a,b^ ± 4.2	2189 ^A^ ± 271.0	9 ^B^ ± 1.7	83 ± 14.4	7 ± 11.0
2014	Spring	13	1502 ^b^ ± 19.4	1029 ^B^ ± 364.3	10 ^b^ ± 4.4	25 ^B,b^ ± 9.7	17 ^A,a^ ± 19.8
	Early summer	8	2419 ^a^ ± 81.8	1360 ^B^ ± 429.2	20 ^a^ ± 8.8	73 ^a^ ± 19.3	3 ^b^ ± 6.8
	Late summer	8	2418 ^A,a^ ± 73.3	977 ^B^ ± 230.6	13 ^A,a,b^ ± 2.6	63 ^a^ ± 22.0	1 ^b^ ± 0.7

* The difference in vegetation cover (%) plus stone cover (%) to 100% is accounted for by bare soil. Small superscript letters (^a,b^) indicate differences between seasons within a year; capital superscript letters (^A,B^) indicate differences for the same season between the two years; *p* ≤ 0.05.

**Table 2:** Concentration of dry matter (DM), organic matter (OM), crude protein (CP), neutral detergent fiber (NDF), and acid detergent fiber (ADF) in herbaceous biomass available along the grazing itineraries of goats on Chinese Altai Mountain pastures. Values depict arithmetic means and standard error of the mean (SEM).

Year	Season	n	DM	OM	CP	NDF	ADF
			(g kg^−1^ FM)	(g kg^−1^ DM)			
2013	Spring	10	522 ^a^	853	136 ^a^	495 ^B^	373 ^a^
	Early summer	6	401 ^b^	881	117 ^b^	479	327 ^A,b^
	Late summer	3	413 ^a,b^	891	126 ^A,a,b^	488	309 ^b^
	*SEM*		*21.6*	*8.1*	*3.5*	*8.3*	*10.4*
	*Effect of season*		*	*n.s.*	*	*n.s.*	*
2014	Spring	13	518 ^a^	858 ^b^	126	571 ^A,a^	388 ^a^
	Early summer	8	382 ^b^	905 ^a^	110	470 ^b^	285 ^B,b^
	Late summer	8	465 ^a^	900 ^a^	114 ^B^	461 ^b^	292 ^b^
	*SEM*		*15.5*	*6.6*	*3.1*	*14.3*	*11.3*
	*Effect of season*		***	**	*n.s.*	***	***
*Effect of year*	*Spring*		*n.s.*	*n.s.*	*n.s.*	**	*n.s.*
	*Early summer*		*n.s.*	*n.s.*	*n.s.*	*n.s.*	*
	*Late summer*		*n.s.*	*n.s.*	**	*n.s.*	*n.s.*

FM = fresh matter, n = number of biomass samples. Differences between seasons within one year are depicted by small letters (^a,b^), while differences between years for a given season are indicated by capital letters (^A,B^). * *p* ≤ 0.05, ** *p* ≤ 0.01, *** *p* ≤ 0.001, *n.s.* = not significant.

**Table 3:** Live weight (LW) of goats grazing on Chinese Altai Mountain pastures, organic matter digestibility (OMD) of the ingested diet and intake of dry matter (DMI), organic matter (OMI), crude protein (CPI), neutral detergent fiber (NDFI), and acid detergent fiber (ADFI). Values depict arithmetic means and standard error of the mean (SEM), *n* = 5.

Year	Season	LW	OMD	DMI	OMI	CPI	NDFI	ADFI
		(kg)	(g kg^−1^ DOM)	(g kg^−0.75^ LW)				
2013	Spring	59	698 ^b^	63	60	8.5	31 ^B^	23
	Early summer	62	722 ^B,a,b^	76	71	9.0	37	25
	Late summer	66	723 ^a^	63	60	7.9	31	19
	*SEM*	*3.0*	*4.8*	*5.3*	*5.0*	*0.63*	*2.5*	*1.8*
	*Effect of season*	*n.s.*	*	*n.s.*	*n.s.*	*n.s.*	*n.s.*	*n.s.*
2014	Spring	60	704 ^b^	76	71	9.5	43 ^A^	29 ^a^
	Early summer	64	756 ^A,a^	73	68	8.0	34	21 ^b^
	Late summer	52	718 ^b^	80	75	9.1	37	23 ^a,b^
	*SEM*	*3.1*	*6.5*	*3.3*	*3.1*	*0.40*	*1.9*	*1.4*
	*Effect of season*	*n.s.*	***	*n.s.*	*n.s.*	*n.s.*	*n.s.*	*
*Effect of year*	*Spring*	*n.s.*	*n.s.*	*n.s.*	*n.s.*	*n.s.*	*	*n.s.*
	*Early summer*	*n.s.*	*	*n.s.*	*n.s.*	*n.s.*	*n.s.*	*n.s.*
	*Late summer*	*n.s.*	*n.s.*	*n.s.*	*n.s.*	*n.s.*	*n.s.*	*n.s.*

Differences between seasons within one year are depicted by small letters (^a,b^), while differences between years for a given season are indicated by capital letters (^A,B^). * *p* ≤ 0.05, *** *p* ≤ 0.001, *n.s.* = not significant.

**Table 4:** Daily fecal dry matter (DM) excretion of goats grazing Chinese Altai Mountain pastures, and fecal concentration of organic matter (OM), nitrogen (N), and neutral detergent fiber (NDF). Values depict arithmetic means and standard error of the mean (SEM), *n* = 5.

Year	Season	DM Excretion		OM	N	NDF
		(g d^−1^)	(g kg^−0.75^ LW)	(g kg^−1^ DM)		
2013	Spring	451	21	834	27 ^b^	619 ^A,a^
	Early summer	493	23	849	30 ^B,a^	546 ^A,a,b^
	Late summer	429	20	837	30^a^	537^b^
	*SEM*	*26.7*	*1.6*	*5.1*	*0.6*	*10.5*
	*Effect of season*	*n.s.*	*n.s.*	*n.s.*	*	**
2014	Spring	527 ^a^	25	852	28 ^b^	548 ^B,a^
	Early summer	427 ^b^	19	858	36 ^A,a^	520 ^B,a,b^
	Late summer	467 ^a,b^	25	853	30^b^	506 ^b^
	*SEM*	*16.2*	*1.3*	*3.9*	*0.9*	*6.3*
	*Effect of season*	*	*n.s.*	*n.s.*	***	**
*Effect of year*	*Spring*	*n.s.*	*n.s.*	*n.s.*	*n.s.*	***
	*Early summer*	*n.s.*	*n.s.*	*n.s.*	***	**
	*Late summer*	*n.s.*	*n.s.*	*n.s.*	*n.s.*	*n.s.*

Differences between seasons within one year are depicted by small letters (^a,b^), while differences between years for a given season are indicated by capital letters (^A,B^). * *p* ≤ 0.05, ** *p* ≤ 0.01, *** *p* ≤ 0.001, *n.s*. = not significant.

## Data Availability

For scientific purposes, data can be made available to individual scientists upon written request to the first author.
